# Development of an in situ assay for simultaneous detection of the genomic and replicative form of PCV2 using padlock probes and rolling circle amplification

**DOI:** 10.1186/1743-422X-8-37

**Published:** 2011-01-24

**Authors:** Sara Henriksson, Anne-Lie Blomström, Lisbeth Fuxler, Caroline Fossum, Mikael Berg, Mats Nilsson

**Affiliations:** 1 Department of Genetics and Pathology, Rudbeck laboratory, Uppsala University, SE-75185 Uppsala, Sweden; 2 Department of Biomedical Sciences and Veterinary Public Health, Section of Virology, Swedish University of Agricultural Sciences (SLU), Uppsala, Sweden; 3 Department of Biomedical Sciences and Veterinary Public Health, Section of Immunology, SLU, Uppsala, Sweden

## Abstract

**Background:**

In this study we utilized padlock probes and rolling circle amplification as a mean to detect and study the replication of porcine circovirus type 2 (PCV2) in cultured cells and in infected tissue. Porcine circovirus type 2 is a single-stranded circular DNA virus associated with several severe diseases, porcine circovirus diseases (PCVD) in pigs, such as postweaning multisystemic wasting syndrome. The exact reason and mechanisms behind the trigger of PCV2 replication that is associated with these diseases is not well-known. The virus replicates with rolling circle replication and thus also exists as a double-stranded replicative form.

**Results:**

By applying padlock probes and rolling circle amplification we could not only visualise the viral genome but also discriminate between the genomic and the replicative strand in situ. The genomic strand existed in higher numbers than the replicative strand. The virus accumulated in certain nuclei but also spread into the cytoplasm of cells in the surrounding tissue. In cultured cells the average number of signals increased with time after infection.

**Conclusions:**

We have developed a method for detection of both strands of PCV2 in situ that can be useful for studies of replication and in situ detection of PCV2 as well as of DNA viruses in general.

## Background

Padlock probes are single-stranded linear oligonucleotides that upon recognition of a target sequence can be circularised using a DNA ligase [1]. The two ends of the probe are hybridised juxtaposed on a target molecule and upon perfect hybridisation at the ligation site the ends are enzymatically ligated. The ligated padlock probes can then be used to template a localised rolling circle amplification (RCA) [2], giving rise to long single-stranded DNA molecules that spontaneously coil into ~1 μm sized objects that can easily be observed as discrete bright fluorescent spots at the sites in cells where they have been generated [3]. The rolling circle products (RCPs) remain covalently linked to the target molecule by using the target strand as a primer for RCA. The technology is suitable to quantify molecules in cells using a for the purpose developed program Blobfinder [4]. Padlock probes and RCA has for example previously been used in situ to genotype single nucleotide polymorphisms in mitochondrial DNA [5] and mRNA [6]. Padlock probes and target primed RCA was also used for in situ detection of *Anaplasma phagocytophilum *and *Anaplasma marginale *infections in cultured cells [7].

Padlock probes and RCA was used in the present study to investigate active replication in porcine circovirus type 2 (PCV2) infected lymph nodes collected from naturally and experimentally infected pigs and in a porcine cell line infected with PCV2. PCV2 is a small non-enveloped single-stranded circular DNA virus in the family *Circoviridae *[8,9]. Although the common prevalence of PCV2 in pigs, it is associated to several diseases in pigs (PCVD) such as postweaning multisystemic wasting syndrome (PMWS), porcine dermatitis and nephropathy syndrome and different reproductive disorders. PMWS is a global disease that occurs in almost all parts of the world and can give large economic losses [10,11]. The main clinical sign of PMWS is wasting but other symptoms such as diarrhoea and dyspnea are common. The lymph nodes of the infected pigs become enlarged and one of the diagnostic criteria is the presence of moderate to high amounts of PCV2 in the tissue. Virus can be found in a number of cells and in pigs with PMWS the virus seem to accumulate in histiocytes, where the virus can be found both in the nucleus and in the cytoplasm [12]. If this is associated with viral replication in these cells and tissue is unclear. Viral replication occurs with rolling circle replication using a double stranded replicative form (RF) as template. The exact mechanism is not yet known but it has been studied extensively in both PCV1 and PCV2 [13].

In this study we have applied padlock probes and RCA to investigate the suitability of the technology for analyses of PCV2 infection in both fresh frozen tissue sections of lymph nodes from experimentally infected pigs, and from pigs suffering from PMWS. Furthermore, the course of PCV2 infection in PK-15A cells was followed for 72 h to show the accumulation of the genomic as well as the replicative strand. The results from this study clearly shows that the technique allowed detection of both the genomic strand and the replicative strand of PCV2 in cultured cells and fresh frozen tissue sections. Thus, the method opens up for further studies of PCVDs in situ.

## Results

**Figure 1:**
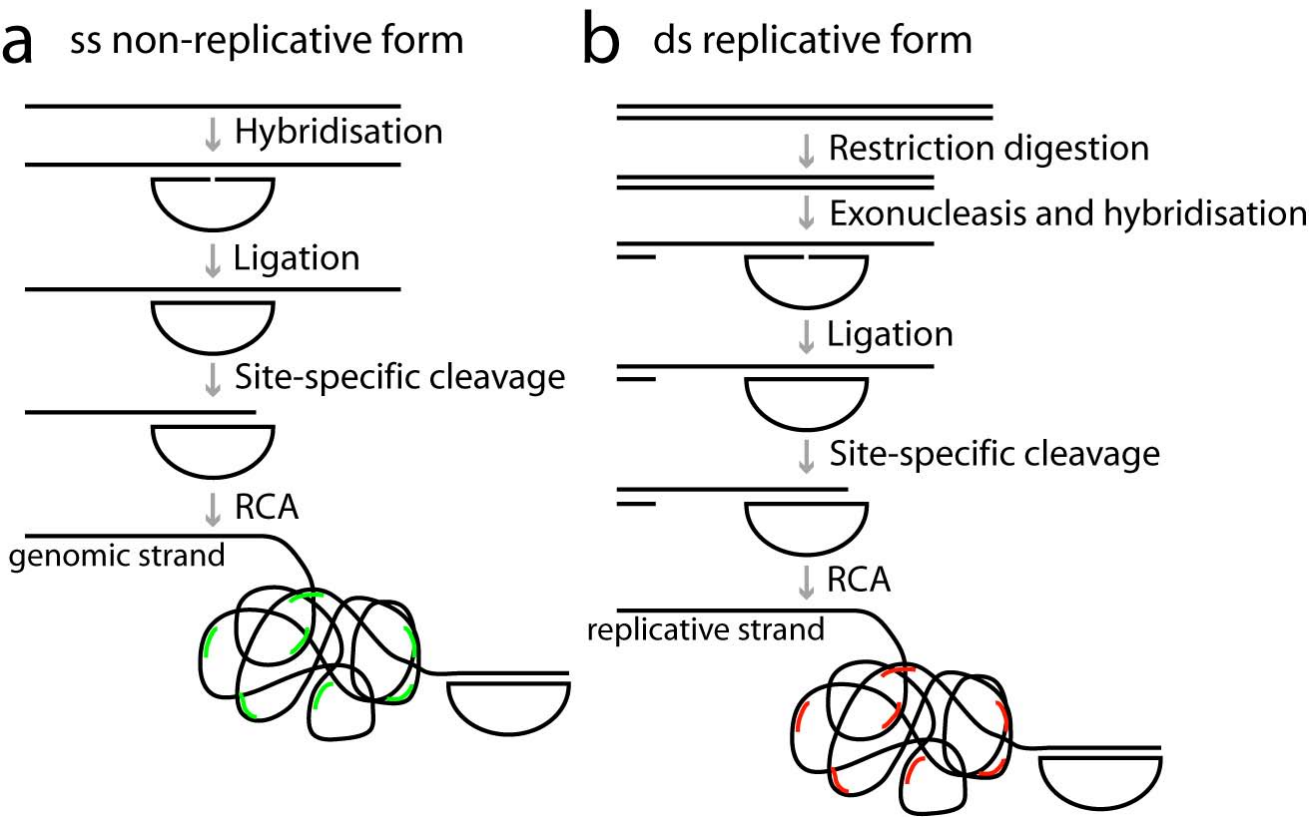
**Schematics over the padlock probe and rolling circle amplification procedure**. Strand-specific padlock probes are hybridised and ligated a) directly on single stranded DNA (for the genomic form), and b) after restriction digestion and exonucleolysis on double stranded DNA (for the replicative form). The target DNA is then cut site-specifically using the combined action of MutY and EndoIV enzymes to create a free 3'-end as a starting point for RCA. After RCA the rolling circle products are visualised by hybridisation of fluorescently labelled detection oligonucleotides, green for the products from the probe specific for the genomic strand and red for the probes specific for the replicative form.

We designed strand-specific padlock probes for both strands of PCV2 in order to distinguish between the genomic and the replicative strands. The two target sites were located in nearby but not overlapping sequences. Since it was unclear to what extent the viral DNA of the replicative form would be double- or single-stranded in cells, we prepared the target DNA such that it would enable detection of both double stranded and single stranded DNA. To ligate and amplify padlock probes, the target DNA has to be single-stranded with a free 3' end where RCA can be initiated (Figure 1). Double stranded DNA was, therefore, cut with a restriction enzyme and made single-stranded with an exonuclease. (Figure 1b). To create a free 3' end in single-stranded target DNA, the DNA was cut site-specifically at a deliberately introduced AG mismatch between the padlock probe and the target sequence (Figure 1b). The mismatch is recognised by MutY, removing the A base in the target sequence, and the phosphate backbone is then cleaved using EndoIV [14]. As we used it here, the method can not tell if the DNA is double or single stranded but only discriminate between the two strands of DNA. However, by omitting the restriction digestion step, one could potentially determine whether the replicative form exists in a single-stranded form.

### Infection of PCV2 in PK-15A cells

**Figure 2:**
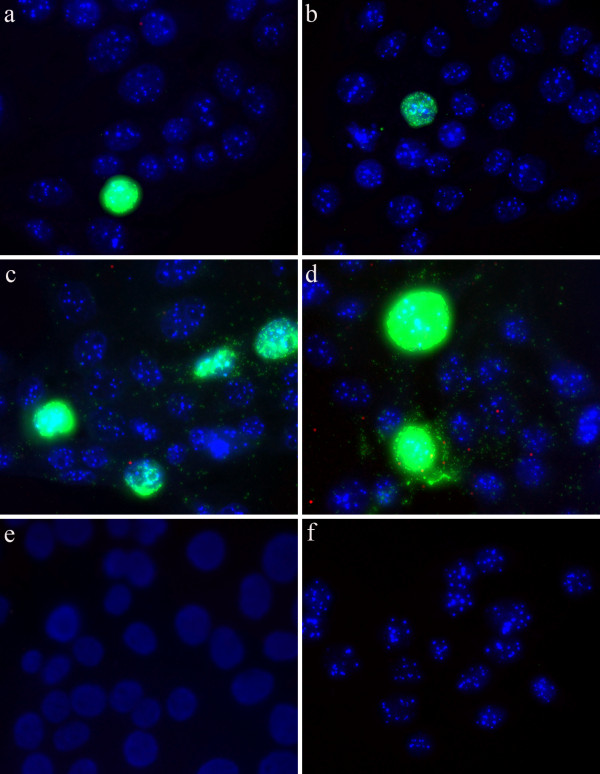
**PCV2 Infection in PK-15A cells**. Path of infection in PCV2 infected PK-15A cells after a) 24 hours, b) 30 hours, c) 48 hours, d) 72 hours, e) 72 hours unligated padlock probe and f) uninfected for 24 hours. Rolling circle products from the genomic strand are seen as green dots, signals from the replicative strand as red dots, and nuclei are counterstained with DAPI (blue).

**Figure 3:**
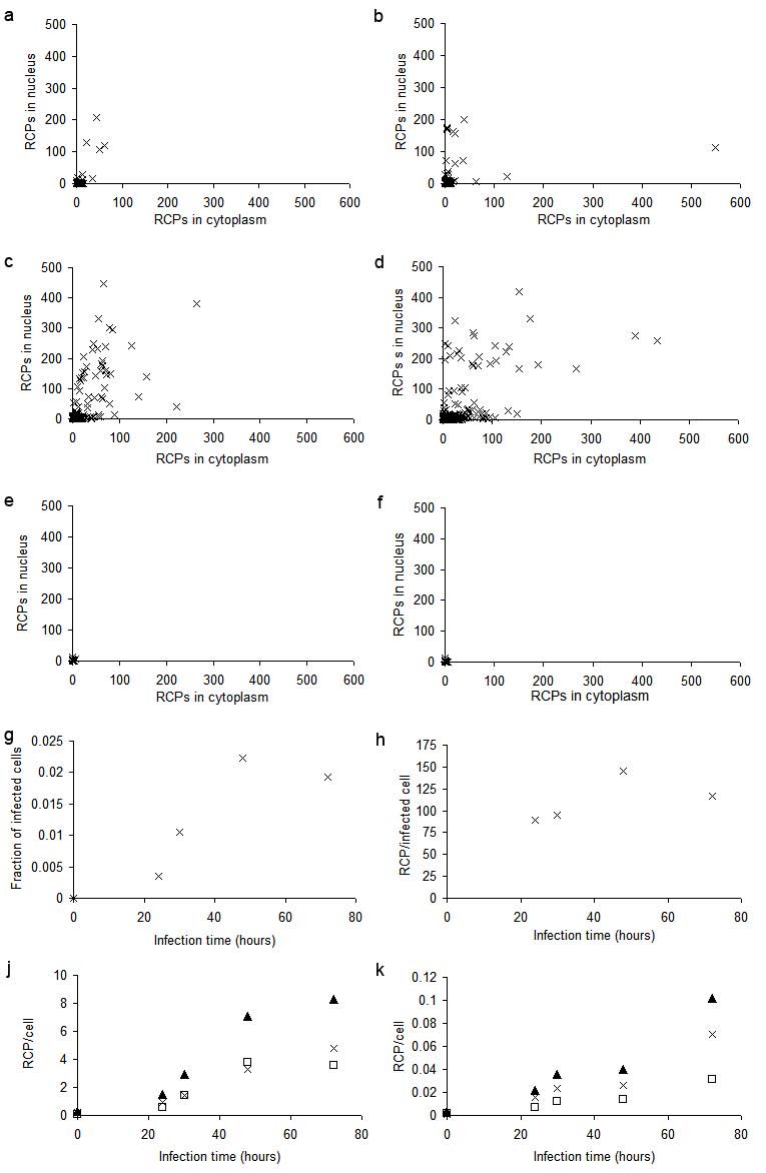
**Quantification of PCV2 infection in cells**. PCV2 infection in PK-15A cells. a-f) Distribution of virus strand RCPs in nucleus and cytoplasm in individual cells at a) 24 h, b) 30 h, c) 48 h, d) 72 h, and e) 0 h after infection. f) negative control without ligase at 72 h after infection, g) fraction of cells with at least 15 RCPs in the nucleus, h) average number of RCPs in cells with more than 15 RCPs in the nucleus, j) average number of RCPs in nucleus are seen as white squares, in cytoplasm as crosses and totally per cell as black triangles from the padlock probe targeting the genomic strand and, k) average number of RCPs in nucleus are seen as white squares, in cytoplasm as crosses and totally per cell as black triangles from the padlock probe targeting the replicative strand.

PK-15A cells were infected with PCV2 for 24, 30, 48 or 72 hours to study the time course of the accumulation of genomic and replicative forms of PCV2. An uninfected control was also included. The infection was performed on triplicate slides. We applied padlock probes and RCA on the cells, including another negative control which had no ligase in the ligation step on cells that were infected for 72 hours. The RCPs from the genomic strand was labelled with FITC and could be seen as green signals and the RCPs from the replicative strand was labelled with Cy3 and could be seen as red signals. The number of signals from the two padlock probes was quantified in the nuclei and in the cytoplasm of individual cells in twenty images from each slide of the triplicates using the Blobfinder software. In infected cells there were signals from both the genomic and the replicative strand already 24 hours post infection. In the negative controls, which were uninfected or lacked ligase in the ligation step, there were very few unspecific signals from the padlock probes (Figure 2) (Figure 3a-f).

There were, as expected, considerable more signals from the genomic strand than from the replicative strand throughout the infection. The signals from the two strands also differed in localisation. The genomic strand was concentrated to a limited number of cell nuclei, which on the other hand had very high numbers of signals. These cells also had a significant number of signals for the genomic strand in the cytoplasm. Abundant signals were also observed in the cytoplasm of neighbouring cells, indicating a recent infection of these cells. At the longer incubation times, most cells had at least some signals from the genomic strand in the cytoplasm. The pattern displayed where certain nuclei were heavily infected, was similar to that shown by immune peroxidase labelling of the cultures for the capsid protein of PCV2 (data not shown).

The signals from the replicative strand were more evenly spread in the cell than the signals from the genomic strand and were present both in the nucleus and in the cytoplasm. They were mostly detected in the cells with very high levels of signals from the genomic strand.

When following the infection of PCV2 for 72 hours we observed, as expected, that the total average number of signals per cell increased over time for both strands (Figure 3j-k). We observed an apparent slight decrease in the number of genomic strands in the nucleus at 72 hours compared to 48 hours, but that could be due to a saturation effect when counting the signals using the RCP counting software. If the signals are very densely packed they tend to merge, resulting in an apparently reduced number of RCP counts.

To see if the proportion of cells that were infected changed over time we defined an infected cell as a cell with 15 or more signals from the genomic strand in the nucleus. The fraction of infected cells increased up to 48 hours and then decreased at 72 hours (Figure 3g). The number of signals in the infected cells also increased up to 48 hours but decreased at 72 hours (Figure 3h).

Using padlock probes and RCA it was also possible to study the proportions of the different strands in different compartments (nucleus and/or cytoplasm) over a time course (Figure 3j - k).

### Localisation of virus compared to Mitotracker staining

**Figure 4:**
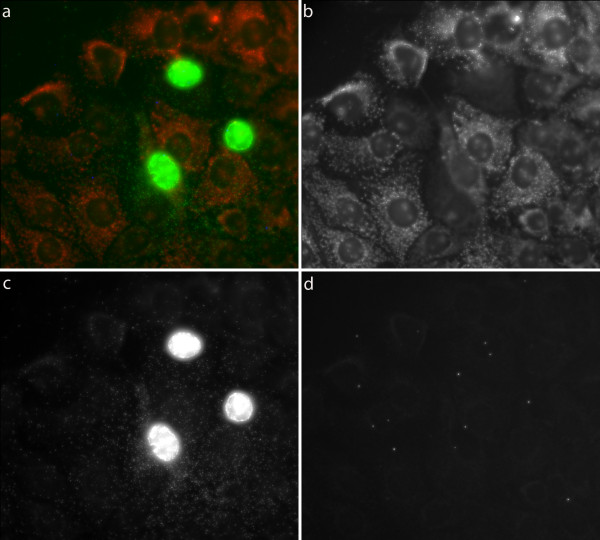
**PCV2 detected in cells with mitochondrial staining**. PCV2 detected in cells with mitochondrial staining. a) Merged image with RCP detecting virus as green dots, replicative strand as blue dots and mitochondria labelled with Mitotracker seen in red, b) Mitotracker, c) signals for the genomic strand and d) signals for the replicative strand.

It has been proposed that PCV2 replicates and is localised in the mitochondrion [15]. We tested this by staining cells with the mitochondrial membrane dye Mitotracker before applying padlock probes and RCA. However, it did not appear like signals from neither PCV2 genomic strand nor the replicative strand colocalized with mitochondria (Figure 4).

### PCV2 detected in tissue samples

**Figure 5:**
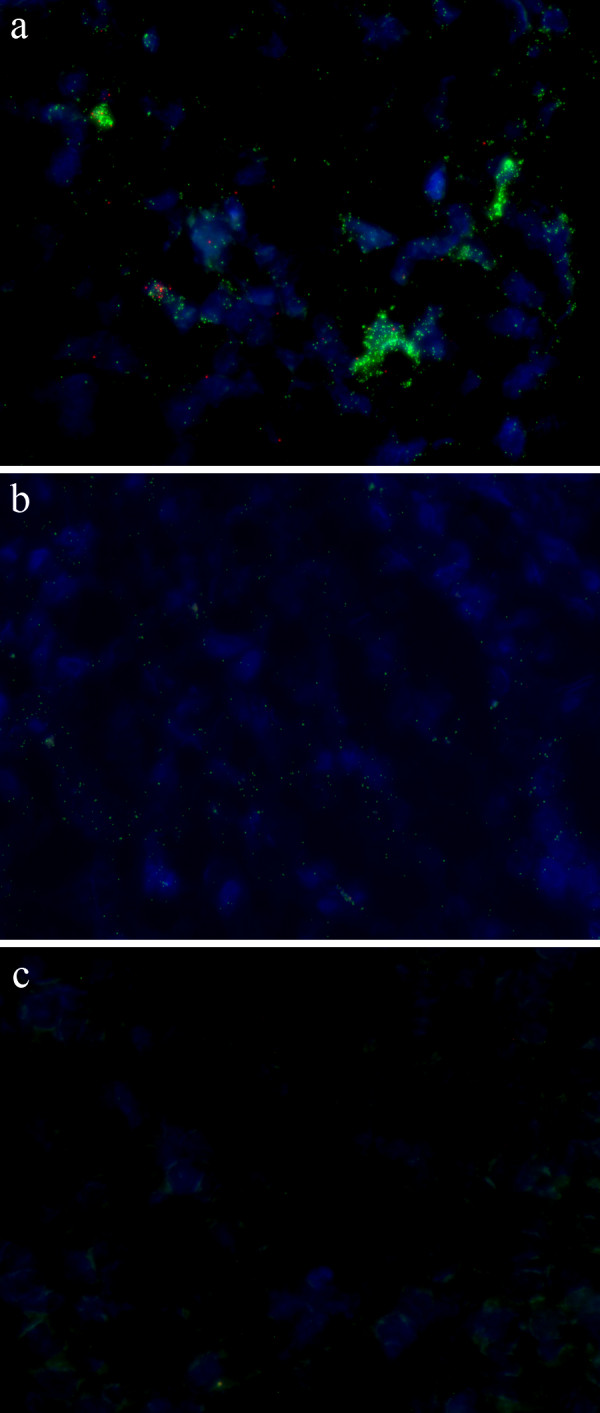
**Detection of PCV2 in lymph node tissue**. Detection of PCV2 with padlock probes in lymph node tissue from a) an experimentally infected pig, b) a natural case of PMWS and c) PCV2 negative tissue. Rolling circle products detecting the genomic strand is seen as green dots, the replicative strand is seen as red dots and the nuclei are counterstained with DAPI (blue).

Padlock probes were further used to detect PCV2 in fresh frozen lymph node tissue sections from both experimentally infected pigs and from a natural case of PMWS (Figure 5). Tissue samples that have been tested negative for PCV2 by qPCR were included as negative controls. Signals were observed from both the genomic and the replicative strand in the tissue from the experimentally infected pig while they were absent in the tissue from the uninfected pig. The majority of the signals were from the probe detecting the genomic strand and they were concentrated in certain cell nuclei, similar to what was seen in the PCV2 infected cell lines. It was only in and around these highly infected cells that signals from the replicative strand were seen. The signals from the replicative strand were located in the nuclei as well as in the cytoplasm. In the natural case of PMWS we only observed signals from the genomic strand and these were spread throughout the tissue section, and thus not densely concentrated to a few nuclei as was seen in the experimentally infected cells and tissues that were in an experimentally induced acute phase of PMWS. However, although this study shows the usability of this technique to detect and study PCV2 replication in tissue, only a low number of pigs were investigated and a more comprehensive study is needed in order to achieve a better understanding of the infection and replication of PCV2 in pigs.

## Discussion

During the early course of PCV2 infection the genomic DNA can be found in the circulation before accumulating in cells of the monocyte/macrophage lineage [16,17]. The site of active PCV2 replication that leads to PMWS and the triggering factors are however not well documented. PCV2 is a single-stranded circular DNA virus, but during replication of PCV2 it exists as a double stranded replicative form. The cellular localization of PCV2 has been studied by electron microscopy of lymph nodes and during the course of infection of a porcine lymphoblastoid cell line, combining ultrastructural analyses with immunogold labelling for the capsid protein of PCV2 [15,18,19]. To enable studies of PCV2 localisation and replication in detail however new methods are necessary. Therefore we designed an assay based on padlock probes and RCA that are able to detect and discriminate between the genomic and the replicative strand of PCV2 in situ. The assay was successfully applied to detect both strands of PCV2 in cells as well as in fresh frozen tissue sections.

PK-15A cells were infected with PCV2 and we followed the infection for 72 hours to study the replication process. The signals for both the genomic and the replicative form increased over time. The signals from the genomic strand were concentrated in certain nuclei but eventually spread to the cytoplasm in all cells. The replicative form was only observed in and around these highly infected cells, indicating that this is the place of replication. The replicative strand was found both in the nucleus and in the cytoplasm of the replicating cell. Though the total number of signals from the genomic strand increased throughout the infection, the fraction of infected cells decreased between 48 and 72 hours. This could be because at 72 hours the cells have started to undergo apoptosis resulting in loss of cells.

Earlier observation [15,18,19] pointed to that PCV2 proteins were associated with mitochondria, and that the mitochondria were suggested to be important for viral replication. Therefore we used the newly developed method to study if we could detect a co-localisation of the genomic and/or the replicative strand with mitochondria stained by Mitotracker. The infected cells had weaker mitochondrial staining from the Mitotracker dye than the uninfected cells. Because Mitotracker stains active mitochondria that have normal membrane potential these differences indicated that infected cells were under stress. However, no correlation between the localisation of mitochondria and viral DNA for any of the PCV2 strands was detected and could not support the findings by Rodriquez-Carino et al. (2009, 2010a, b). However, our studies were not very comprehensive and needs to be repeated to be clear on this issue. One obvious difference is that we detect viral DNA while they detect viral proteins. It is possible that viral proteins localise in the mitochondria and perform some function there. Furthermore, our labelling was conducted on cells after 48 h of infection whereas the co-localisation of PCV2 cap protein with mitochondria was most obvious during the earlier phase of infection [19]. Another potential explanation for these discrepant results would be if the viral DNA is more strongly associated with viral proteins in the mitochondrion than elsewhere in the cell and thus less available for probing.

We also applied the technique on lymph node tissue from an experimentally infected pig and from a pig suffering from PMWS. In the experimentally infected tissue we found signals from both strands but the signals from the padlock probe targeting the genomic strand were much more abundant compared to that of the probe targeting the replicative strand, showing that the genomic strand is present in much higher levels than the replicative strand also during early phases of infection. In the naturally infected tissue we only observed signals from the genomic strand, possibly indicating that PCV2 had ceased to replicate.

Different methods have been used by others to detect PCV2 in situ, such as immunohistochemistry, and in situ hybridisation [20-22]. Padlock probes can distinguish between different isolates that only differ at a single base, which is not possible with FISH or in situ PCR. Padlock probes also have an advantage of being able to discriminate between the two complementary strands which is otherwise difficult with FISH or when studying viruses with PCR. In situ detection allows for intra cellular localisation of the two complementary strands and can thus be used to study the path of infection. A combination of FISH using probes detecting PCV2 and fluorescence immunohistochemistry (FIHC) with fluorescent antibodies to single stranded or double stranded DNA has been elaborated and applied on tissue sections from gnotobiotic pigs [23], but with that technique other DNA strands than those from PCV2 will also be detected if present. With padlock probes it would also be possible to demonstrate the presence of different viruses in multiplex by using various combinations of fluorophores for detection. In this study we choose to study PCV2 in fresh frozen tissue sections and in cultured cells, but this method could be used for in situ studies of single-stranded DNA viruses in general. Especially interesting would be to study the known co-infection of PCV2, torque teno virus and porcine bocavirus in situ [24].

## Conclusions

We developed an in situ assay for the simultaneous detection and discrimination of the genomic and the replicative form of PCV2, in cells and lymph node tissue from pigs using padlock probes and RCA. This assay can be used, as shown in this paper, to study different aspects of viral replication of PCV2. Even though we here chose to study PCV2 this method could be used to study other DNA viruses as well as with some modifications RNA viruses.

## Methods

### Design of padlock probes

Thirty nucleotide long sequences were selected in the viral genome of PCV2 as target sequences. The sequences were chosen so that they would target 28 known Swedish isolates of PCV2 [25] and padlock probes for both strands of PCV2 were designed accordingly genomic strand padlock probe: AGAATAAGAAAGGTTGCGACTATCTAT*CCTCAATGCTGCTGCTGTACTAC*TTCCTCCCGTTGAATACTAC and replicative strand padlock probe: GTAACGGTGGCGGGGTTCCTTTTACGA*AGTCGGAAGTACTACTCTCT*TTCTACGATTGCCTTCTCCAGCG. The two target sequences were located on the same ORF close to each other but they were not overlapping to avoid hybridisation between the two padlock probes. The padlock probes contained target complementary sequences in the ends and a sequence for detection in the middle (italics). The same sequences were used with a 5' fluorophore for detection. The padlock probes also contain a mismatch (underlined) for site specific cleavage by MutY and EndoIV [14].

### Cells and tissues

Approximately 10^5 ^PK-15A cells per ml DMEM supplemented with antibiotics and 4% FCS were grown in Leighton tubes on 9 × 40 mm cover slips until one third of the area was covered. Cells were incubated together with PCV2 (PCV2 Imp.1010-Stoon; 5 x10^3 ^TCID_50_) for five hours before carefully rinsed and incubated in Leighton tubes with 2 ml fresh medium. Cells were then fixed in ethanol after a total infection time of 24, 30, 48 and 72 hours. Parallel cultures were grown with 100 nM Mitotracker^® ^Orange CMTMRos before fixation after 48 h. Negative control slides were also prepared with non-infected PK-15A cells grown for 24 h before fixation. All slides with cells were prepared in triplicates. Tissue sections, 10 μm thick, were prepared from archived material, *i.e.*, fresh frozen lymph nodes stored in liquid nitrogen. The lymph nodes were collected from a pig experimentally co-infected with porcine parvovirus and PCV2 terminated 28 days post infection, from an uninfected control pig [26], and from a clinical case of PMWS where time of infection is unknown but having PCV2 DNA in its blood a month before termination [27]. Tissue slides were kept dry in a -70°C freezer until used.

### Target preparation

The slides with cells or tissue sections were treated with 0.01% pepsin in 0.1 M HCl for 90 or 270 seconds respectively and then washed with phosphate buffered saline. Slides with cells were dried in a series of ethanol 70, 85 and 99.5% and Secure Seals (SA16S-0.5; Grace Bio-labs) were then attached to the slides. All the following reactions on cells were performed with ~40 μl within a 7.0 × 7.0 × 0.8 mm Secure Seal, and all reactions on tissues were performed in a reaction volume of 20 μl under a 22 × 25 mm LifterSlip (22I × 25-2-4635; Erie Scientific Company), except for the RCA step which was performed in a volume of 40 μl within a circular area defined using an ImmEdge Pen (Vector Laboratories). All the reactions were performed in a humid chamber and all washes were performed in either the Secure Seal, or in a coupling jar. Secure Seals were rinsed with TBS supplemented with Tween-20 to minimise formation of air-bubbles in the following reactions. DNA was restriction digested and made single-stranded in a combined reaction with 0.5 U/μl NlaIII and 0.4 U/μl T7 exonuclease (New England Biolabs) in 1x NEB4 buffer supplemented with 0.2 μg/μl BSA (New England Biolabs). The reaction was carried out at 37°C for thirty minutes and cells and tissues were then washed in 0.1 M Tris- HCl, 0.15 M NaCl, and 0.05% Tween-20.

### Hybridisation and ligation of padlock probes

One hundred nM of each padlock probe (Integrated DNA Technology) was hybridised to the target sequences and ligated with 0.25 U/μl Ampligase (Epicentre) in Ampligase buffer supplemented with 0.2 μg/μl BSA, 1 mM NAD, 125 mM KCl, and 5% glycerol. The reaction was carried out at 55°C for one hour. Cells and tissues were then washed in 2x SSC and 0.05% Tween-20 for five minutes at 37°C and then in 0.1 M Tris- HCl, 0.15 M NaCl, and 0.05% Tween-20.

### Site specific cleavage with MutY and EndoIV

The padlock probes contained an AG mismatch. The A in the target sequence was cleaved off with MutY and EndoIV to produce a free 3' end as a starting point for RCA [14]. Two μM MutY (usb) and 0.2 U/μl Endo IV (Fermentas) was used to cut single-stranded DNA in a reaction with 0.5 μg/μl BSA, 20 mM Tris-HCl (pH 7.6), 30 mM NaCl, 1 mM EDTA, 0.1 M KCl, and 1 mM DTT at 37°C for thirty minutes. Cells and tissues were then washed in 0.1 M Tris- HCl, 0.15 M NaCl, and 0.05% Tween-20.

### Rolling circle amplification

Circularised padlock probes were amplified with RCA. Phi29 DNA polymerase (Fermentas) was used in the supplied buffer supplemented with 0.2 μg/μl BSA, 0.25 mM dNTP, and 5% glycerol. RCA was performed for one hour at 37°C. Cells and tissues were then washed in 0.1 M Tris- HCl, 0.15 M NaCl, and 0.05% Tween-20.

### Detection and mounting

Two hundred and fifty nM of fluorescently labelled detection oligonucleotide was hybridised to the RCP in a reaction containing 20% formamide and 2x SSC at 37°C for fifteen minutes. Cells and tissues were then washed in 0.1 M Tris- HCl, 0.15 M NaCl, and 0.05% Tween-20. Slides were then dried in ethanol and Secure Seals were removed. Nuclei were counterstained with 100 ng/mL DAPI in Vectashield mounting media (Vector laboratories). Cover slips with cells were mounted onto a microscope slide and microscope slides with tissue sections were covered with 22 × 22 mm cover slips.

### Microscopy

Slides with cells or tissue sections were studied with an Axioplan II Zeiss with 40x magnification and images were collected with the Axiovision 4.5 software. DAPI was acquired for 150 ms for cells and 50 ms for tissue, FITC for 100 ms and Cy5 for 800 ms. Twenty images with 11 z-stacks with 0.495 nm between two z-stacks were taken from each slide of the triplicates with cell cultures and the images were then analysed with the Blobfinder software [4].

## Competing interests

MN owns shares in the company Olink AB that holds the commercial rights to the technique.

## Authors' contributions

SH designed padlock probes, performed padlock and RCA experiments and analysed data with the Blobfinder software. ALB and LF performed PCV2 infections in cells. SH, ALB, CF, MB, and MN planned the study. MB and MN supervised the work. All authors have contributed to the writing of the manuscript and have read and approved the final manuscript.
